# Survey of *Toxocara* eggs on Dog Hair as a Potential Transmission Route in Human Toxocariasis in Northeastern Iran

**Published:** 2020

**Authors:** Neda SASANNEJAD, Javad KHOSHNEGAH, Amin BAKHSHANI, Hassan BORJI

**Affiliations:** 1. Department of Pathobiology, Faculty of Veterinary Medicine, Ferdowsi University of Mashhad, Mashhad, Iran; 2. Department of Veterinary Clinical Science, Faculty of Veterinary Medicine, Ferdowsi University of Mashhad, Mashhad, Iran

**Keywords:** *Toxocara canis*, Eggs, Hair, Feces, Dog

## Abstract

**Background:**

*Toxocara canis* is a gastrointestinal nematode of dogs and other canids with high zoonotic potential. Human infection occurs following ingestion of infective eggs that have been passed in the dogs’ feces. Contact with contaminated soils, is one of the most important risk factors for human infection by *T. canis* eggs. However, in recent studies transmission of infective eggs, through human contact with contaminated dogs’ hair have been proposed. The aim of the present study was to assess the prevalence of *Toxocara* eggs on the hair and feces of dogs which attended to Veterinary Clinic of Ferdowsi University of Mashhad, Iran.

**Methods:**

A total 100 dogs which attended to the clinic were used in the present study. The hair samples were collected from the head, back and perineal region of dogs’ body. Besides collecting hairs, fecal samples were also collected and analyzed for the presence of *T. canis* eggs.

**Results:**

*T. canis* eggs were found in 11% of the hair samples and 10% of the feces samples. Additionally, it has been observed that the risk factors impact such as breed, season of sampling, sex, hair length, indoor-outdoor access and age, were not significant on the *T. canis* eggs presence in the faecal and hair samples.

**Conclusion:**

Human exposure to the hair of dogs, may be significant risk factor for infection and regular anthelmintic treatment, hygiene of animals and public education of the importance of dogs are recommended to prevent human toxocariasis.

## Introduction

T*oxocara canis* is a gastrointestinal helminth of dogs and other canids with high zoonotic potential (1). Adult nematodes reside in the small intestine of definitive hosts and passing its eggs in the feces. Human infection occurs following ingestion of infective eggs. After ingestion, the larvae hatch from eggs and migrate to different organs, causing visceral larva migrants (VLM), ocular larva migrants (OLM), covert toxocariasis (CT), neurotoxocariasis (NT) and common toxocariasis(2).

Contact with potentially contaminated soils with dog faeces is one of the most important risk factors, especially in children due to their soil-consumption behavior. Due to the fact that eggs require a maturation period of time after egestion from dogs, close contact with dogs, not considered a risk (3–5). However, in recent studies transmission of infective eggs, through human contact with contaminated dog’s hair have been proposed. The presence of infective eggs in the coat of dogs further implicates physically direct contact as important transmission route (6–8).

There is only one study reported so far in north-west of Iran (Urmia), that evaluated hair contamination of dogs, and the prevalence was reported to be 36.2% (9). The objective of present study was to investigate the prevalence of *Toxocara* eggs on the hair and feces of dogs which attended to veterinary clinic of Ferdowsi University of Mashhad and to evaluate risk factors for the presence of the eggs such as breed, age, season of sampling, sex, hair length, and indoor-outdoor access.

## Materials and Methods

From February 2017 to January 2018, a total of 100 dogs which attended to veterinary clinic were used in the present study. The average weight of the hair samples collected was 0.45 g. Besides collecting hairs, fecal samples (1g) of dogs were also collected and analyzed for the presence of *T. canis* eggs. For detection of *T. canis* eggs from the hair samples, Wolfe and Wright modified method was used (8).

Statistical analyses were carried out using Chi-square test of the SPSS software version 22 for windows (SPSS Inc., Chicago, IL, USA) for qualitative data and Kruskal–Wallis test for qualitative data. A *P*-value <0.05 was considered statistically significant.

## Results

*T. canis* eggs were found on the hair of 11 (11%) dogs which attended to veterinary clinic of Ferdowsi University of Mashhad over a period of 12 months (Table 1).

**Table 1:** *Toxocara canis* eggs in faces and in hair of 11 egg positive dogs

***Breed***	***Age**^a^***	***Sex***	***Outdoor or Indoor Access***	***Hair length**^b^***	***Eggs in Faeces**^c^***	***Eggs in hair***	***Month***
Afghan	3m	Female	Out	S	+	+	June
	3y	Male	Out	S	+	+	December
Terrier	11m	Female	Out	L	+	+	October
	8m	Male	In	L	−	+	February
	42m	Male	In	L	+	+	March
Dachshund	11m	Female	In	S	+	+	October
Shih Tzu	15m	Female	In	L	+	+	October
	14m	Male	In	L	+	+	March
Corgi	1.5 y	Male	Out	S	+	+	February
Welsh Corgi	1y	Male	Out	S	+	+	March
Spitz	11m	Female	Out	S	+	+	October

a: m: months, y: years.

b: S:short,L:long

c: (+) presence of *T. canis* eggs

Among 11 egg positive dogs, 2 (18%) were puppies, 5 (45%) were juvenile and 4 (37%) were adult dogs. The sex ratio was 5(46%) females and 6 males (54%). 6 short-hair and 5 long-hair dogs had eggs present in their hair samples. Concerning season, the dog infection in autumn (36.3%) was higher compared with the summer (9.09%), spring (27.27%) and winter (27.27%). Moreover, among the infected dogs, 6 (46%) were of indoor access and 4 (54%) were indoor access dogs. Breed, age, season of sampling, sex, hair length, and indoor-outdoor access effects were not significant on the *T. canis* eggs presence in the hair samples (Table 2). The mean eggs per gram (E.P.G) found on hair samples was 5.86 ± 4.20 with a median of 2. The mean eggs from the perianal, back and head regions were 11.80 ± 1.73, 3.37 ± 0.89 and 4.70 ± 6.23 respectively. In addition, the mean of E.P.G found on the different age groups were 9.77 ± 1.43 in puppies, 5.84 ± 1.72 in juvenile and 4.33 ± 0.28 in adults (Table 2). However, the difference between E.P.G mean on different parts of the dog’s body and age group were not significant.

**Table 2:** Densities of *Toxocara canis* eggs per gram of hair and classification observed on the hair of dogs from Northeast of Iran

	***No. Of egg positive samples***	***Location on coat***	***Mean E.P.G. ±SD***	***Median E.P.G.***
Puppy	2 (18%)	Perianal	17.11 ± 2.10	15
		Back	4.90 ± 0.91	4
		Head	7.30 ± 3.53	8
		All sites	9.77 ± 1.43	4
Juvenile	5 (45%)	Perianal	10.41 ± 2.57	9
		Back	2.91 ± 0.45	2
		Head	4.20 ± 2.22	3
		All sites	5.84 ± 1.72	3
Adult	4 (37%)	Perianal	8.07 ± 3.79	7
		Back	2.25 ± 0.84	3
		Head	2.83 ± 1.46	2
		All sites	4.33 ± 0.28	4
Total	11	Perianal	11.80 ± 1.73	10
		Back	3.37 ± 0.89	4
		Head	4.70 ± 6.23	3
		All sites	5.86 ± 4.20	2

*T. canis* eggs were found in the feces of 10 dogs with a prevalence of 10 %. Based on the result, one of the eggs-positive dogs on the fecal examination, don’t show hair contamination. Among the fecal positive dogs, also breed, age, season of sampling, sex, hair length, and indoor-outdoor access effects on the eggs presence were not significant.

## Discussion

In the current study*, T. canis* eggs were found in 10% and 11% of dogs’ hair and feces samples respectively. This results are in agreement with Overgaauw et al. survey, who found the eggs in the 12.2% of hair sample in the Netherlands (5). In addition, some studies recorded 8.8% of hair contamination in Ireland (4) and 14% in turkey (10). Öge et al (10) found *T. canis* eggs in 5% dogs feces sample. In the Iran study, Tavassoli et al reported higher prevalence of 36.2% of dogs harboring *T. canis* in their hair (9).

The present study found that age not important factor in the *T. canis* contamination. Similarly, some previous studies reported no significant effect of age on the present of the eggs on the hair, In contrast, Roddie et al (7) and Wolfe and Wright (8) found that *T. canis* eggs present in puppies are more than young and adult dogs. These studies show that age of the dog is important risk factor for the presence of *T. canis* eggs. Aydenizöz-Özkayhan et al reported that the age of dog is more contributed to the prevalence of eggs than coat type or dog breed (6).

Our results suggest that there was no significant difference in the risk of hair and feces contamination with *T. canis* related to the sex of the animals. However, 56% of dogs in this study were male. Risk of *T. canis* eggs in male dog’s hair was significantly higher than compared to female dogs (11). They suggest that risk of human infection with *Toxocara* increased by exposure to male dogs. In London intestinal infection in young male dogs was significantly higher than in females’ dogs (12).

According to the results of the present study there is no significant link between prevalence of *T. canis* and hair length. Presence of *Toxocara* spp. eggs in short-haired dogs were higher than long-haired dogs (13). It is suggested that temperature conditions close to the skin in shorter hair, are more favorable for *T. canis* eggs development compared to long hair.

There are limited studies that evaluated dog breed effect on the *T. canis* infection. In the current study we observed more parasite prevalences in retriever breed dogs. Senlik et al (14) found higher prevalence of *T. canis* in Belgian Malinois breed compared with Irish setter, German Shepherds and retriever breed. Prevalences of *T. canis* and other gastrointestinal parasite between defined-breed and mixed-breed was similar. Presence of *T. canis* eggs in both faeces and hairs was same in the different breed (6).

Considering dog outdoor-indoor accesses, *T. canis* eggs in fecal samples of outdoor accesses dogs were greater than indoor dogs. While in hair samples, indoor dogs having more contamination than outdoor accesses dogs. In agreement with this results, Wael et al (11) found that the prevalence of *T. canis* eggs in stray dogs’ hair sample was higher than the domestic dogs, and reported that the domestic dogs are big risk for human infection.

In this survey, the overall mean E.P.G of hair was 5.86. Similar to our finding, Overgaauw et al found a 3.8 E.P.G from dog’s hair in the Netherlands (5). Lower eggs per gram were obtained by Keegan et al (10) and Rojas et al (15) that were reported 0.1 and 1.4 EPG, respectively. A study found 584 EPG in dogs’ hair which is much higher than our results (7), which may be related to the fact that they only examined stray dogs and proposed that poor hygiene condition and lack of anthelminthic treatment caused the high number of *T. canis* eggs. The higher number of *T. canis* eggs in the perianal region might be due to contamination of dog's hair from feces.

## Conclusion

In this study, we confirmed the presence of *T. canis* eggs in both hair and feces of dogs which attended to veterinary clinic of Ferdowsi University of Mashhad, Iran. Indicated that human exposure to the hair of dogs, may be important risk factor for infection. However, studied factors include age, breed, sex, season of sampling, hair length and indoor-outdoor access, were not evaluated as significant risk factors. Regular anthelmintic treatment, hygiene of animals and public education of the importance of dogs are recommended to prevent human toxocariasis.
